# 9-Acridinemethanamine and Acridine-9-Carboxaldehyde as Potential Fluorescence Lifetime pH Indicators

**DOI:** 10.1007/s10895-020-02564-5

**Published:** 2020-06-04

**Authors:** Christian Totland, Peter J. Thomas, Bodil Holst, Naureen Akhtar, Jostein Hovdenes, Tore Skodvin

**Affiliations:** 1 Department of Chemistry, University of Bergen, Allégaten 41, 5007 Bergen, Norway; 2 Present Address: NGI – Norwegian Geotechnical Institute, Sognsveien 72, 0806 Oslo, Norway; 3 NORCE Norwegian Research Centre AS, Fantoftvegen 38, 5072 Bergen, Norway; 4 Department of Physics and Technology, University of Bergen, Allégaten 55, 5007 Bergen, Norway; 5 Aanderaa – a Xylem brand, Sanddalsringen 5b, N-5225 Nesttun, Norway

**Keywords:** pH sensor, Fluorescence lifetime, Acridine, 9-acridinemethanamine, Acridine-9-carbaldehyde

## Abstract

A significant challenge concerning the development of fluorescence lifetime (FL) based pH sensors is the paucity of fluorophores with sufficiently large FL variation with pH. Acridine is amongst the indicators with highest fluoresce lifetime responses to pH, with a change in lifetime of about 13 ns within a pH range of 5–8. Here we examine the two acridine derivatives, 9-acridinemethanamine (9-AMA) and acridine-9-carbaldehyde (9-ACA) in terms of their FL pH sensitivity and pH sensing range. Both indicators are characterized when dissolved in buffer solutions, as well as when immobilized in support materials. 9-AMA has a change in FL of 11 ns between pH 2–5, both when dissolved in solution and when immobilized in surfactant-filled mesoporous silica. The FL of 9-ACA is not sensitive to pH when dissolved in buffer solutions; however, when covalently bound to amine-modified silica, its FL changes 15 ns between pH 3–6. 9-AMA and 9-ACA represent promising FL in the pH range of pH 2–6, and could potentially form the basis of new FL pH sensors.

**Figure:**
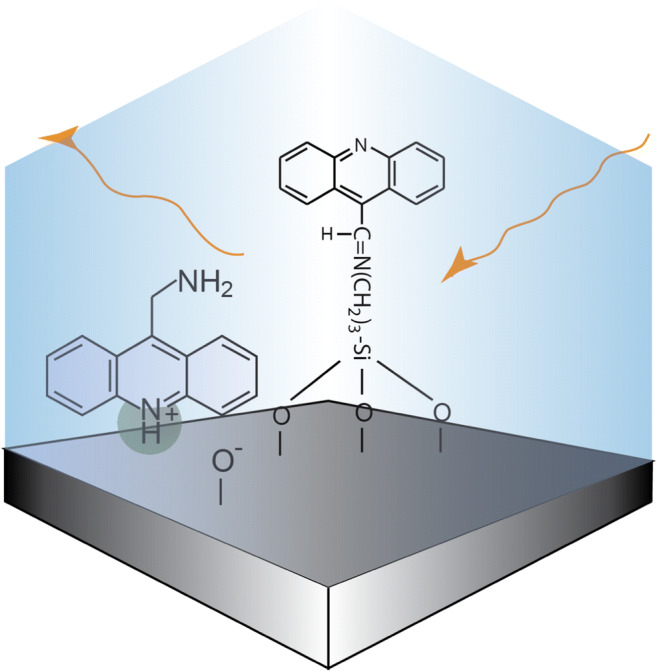
Graphical Abstract

Graphical Abstract

## Introduction

In recent years, the research and development of optical chemical sensor technology has been increasing due to their low cost, low power consumption and good long-term stability [1]. Such characteristics make optical sensors particularly interesting for applications in oceanography [2], aquaculture [3] and biomedicine [4] amongst other applications. Optical pH sensor technologies in particular are attracting a lot of interest not only because of the widespread importance of pH measurements, but also because of the poor long-term stability of commonly available technology such as electrochemical pH probes. Optical pH sensors typically comprise a sensing film consisting of a pH indicator immobilized within an ion penetrable medium. When the pH of the liquid surrounding the film changes, this causes some optical property of the indicator to change, which can be quantified using low cost and robust optoelectronic methods. Some common challenges facing optical pH sensors include photo bleaching from sunlight or probe light from the sensor itself, leaching of the indicator from the immobilizing medium and noise contributions from background luminescence, and probe light intensity variations [5]. Fluorescence lifetime (FL) on the other hand is an intrinsic property of the molecule, and is therefore not affected by these factors.

A significant challenge facing the development of FL-based pH sensors is that very few fluorophores have been identified that exhibit a large change in FL with pH. Sensor architectures revolving around the use of sensor films containing multiple chemical species have been proposed to get around this problem. For example, the dual-lifetime referencing (DLR) method involves a pH sensitive fluorophore together with a long-lived luminescent reference, where the fluorescence intensity of the pH sensitive indicator varies with pH. The combined FL of the indicator pair is much longer and varies over a wider range than the pH sensitive indicator alone making the optoelectronic readout of the FL less challenging [6–8]. Other approaches exploit Föster energy transfers, where a pH insensitive donor with a long luminescence lifetime, such as lanthanide ions, transfers energy to a pH sensitive dye [9–11]. The antenna-mediated effects can cause large changes in lifetime, and thereby has potential for highly accurate pH sensors. However, some sensors with multiple indicators have the disadvantage that the overall measured FL will be influenced if the fluorescence properties of one indicator changes differently to the other (e.g. due to difference in photo bleaching or leaching behavior). For these reasons, a sensor architecture incorporating a single indicator with sufficiently large FL pH variation remains in principle a more attractive option for obtaining long-term calibration stability [12].

Most pH FL indicators, such as fluorescein or fluorescent proteins used for FL imaging [13, 14], only have a 1–2 ns change in FL between high and low pH. This is adequate for lab instruments capable of making high precision FL measurements, but insufficient for achieving a reasonable pH measurement precision in a field deployable pH sensor, where typically only low cost and compact optoelectronics can be used for performing the lifetime measurements. There has been a lot of effort focused towards finding alternative indicators that show higher FL responses to pH change. For example, mercaptopropionic acid-capped quantum dots have been shown to exhibit a FL shift from 8.7 ns to 15.4 ns in the pH range ~ 5 to 8 [15]. In another case, the FL of carbon dots was demonstrated to shift from ~13 ns to 19.5 ns in the pH range 3.4 to 11 [16]. Recently, diazaoxatriangulenium based pH indicators exhibiting a lifetime change from ~7 to 13 ns were reported over the pH range 3 to 8 [17].

Acridine is amongst the indicators with highest fluorescence lifetime responses to pH [18–20]. Acridine derivatives are much studied for their potential applications in cancer treatment [21], or as antibacterial [22] or antiviral agents [23]. With a total change in FL of 20 ns between high and low pH, acridine has potential to match, or even exceed, the accuracy of the electrochemical sensors, even when modest frequency-domain approaches are used for performing the lifetime measurement. However, similar to optical pH indicators in general, the pH sensing range for acridine is limited to 2–3 pH units centered on the pK_a_ of the indicator, i.e. ~pH 5.5–8 [18]. Therefore, it is necessary to find high response FL indicators across a range of pK_a_ values. To date, no other organic FL pH indicators with a sensitivity which matches that of acridine has been proposed. Sensitive indicators that cover the sensing ranges below pH < 6 or pH > 8 are therefore required.

In previous work, it was shown that the pK_a_ of acridine could be shifted to about 9 when immobilized in Nafion [18]. This highlights the importance when selecting the immobilizing medium, which should provide protection for the FL indicator molecule, without adversely influence the sensing characteristics. Mesoporous silica shows particular promise as an immobilizing medium due to high surface area offering potential high indicator loadings for increased signal, and good proton transport properties [20].

**Fig. 1:**
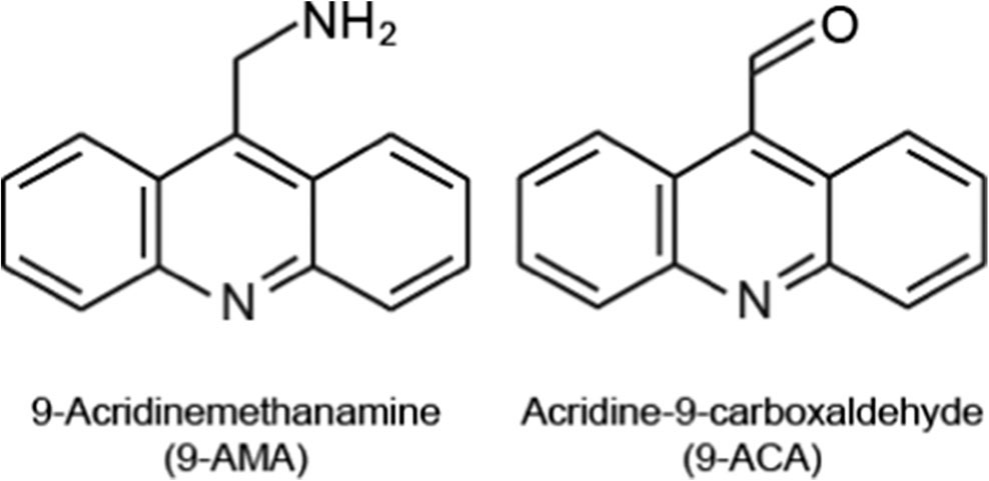
Chemical structures of 9-AMA and 9-ACA

Here we present the pH induced FL response characteristic of two commercially available acridine derived fluorophores, 9-acridinemethanamine (9-AMA) and acridine-9-carboxaldehyde (9-ACA), see Fig. 1, which we show are sensitive in the lower pH ranges from pH 2–6. This pH range is relevant for both physiological applications and industrial applications such as those involving fermentation [24]. Furthermore, we assessed the characteristics of the indicators following immobilization in solid matrices. 9-AMA is immobilized in a surfactant phase within a mesoporous silica, whereas 9-ACA is bound covalently to amine-modified porous silica via the aldehyde group.

## Materials and Methods

### Fluorescence Lifetime Indicators

Acridine (97%), (3-Aminopropyl) triethoxysilane (APTES, 98%), tetraethoxysilane (TEOS, 98%) and acridine-9-carbaldehyde (9-ACA), tetradecyltrimethylammonium bromide (TTAB) was obtained from Sigma Aldrich. 9-acridinemethanamine (9-AMA) was obtained from Fluorochem.

### Immobilization of 9-ACA in Amine Modified Silica Particles

**Fig. 2:**
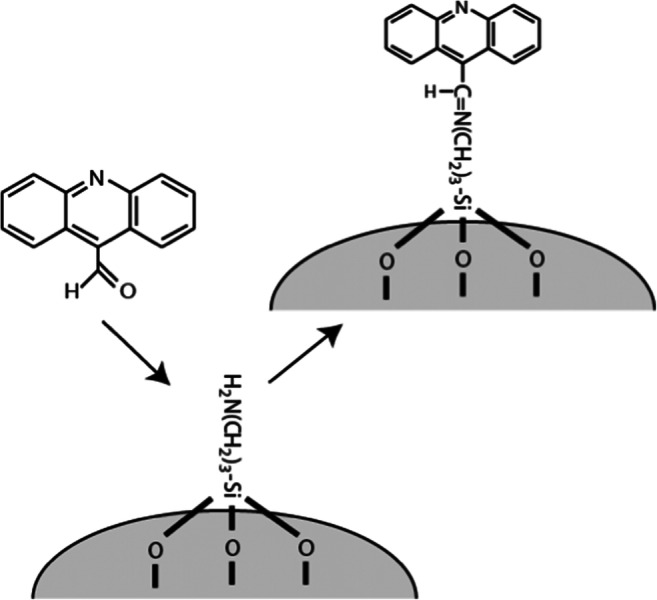
Schematic description of the material synthesized by bonding 9-ACA to amine-modified porous silica

Amine modified silica particles were prepared as described by Chen et al. [25]. In short, TEOS and APTES were mixed in a 1:1 mol ratio. The samples were mixed at room temperature for one hour, after which the opaque suspensions were centrifuged to separate the particles and solution. The particles were washed three times in water and finally vacuum-dried overnight. The dried powders were subsequently suspended in 10 mL of 10 mM 9-ACA in methanol and mixed overnight. The final powder was washed twice with methanol and then once with water before vacuum-drying. The 9-ACA bonds covalently to primary amines via the aldehyde functional group, see Fig. 2.

### Immobilization of 9-AMA in Mesoporous Silica Particles

9-AMA was immobilized according to a procedure we have published previously [20]. In short, a mesoporous silica was synthesized by mixing 2.3 mmoles of TTAB and 0.23 mmoles of 9-AMA in 16.7 mL water, which was gently heated to dissolve the powders completely. After cooling to room temperature, 25.3 mL EtOH and 4.4 mL NH_3_ were added and the solution stirred for 15 min. Then 1.66 mL TEOS was added and the solution stirred for an additional 2 h. The powder was washed four times by shaking with 40 mL water followed by centrifugation to remove the water. The powder was finally dried at 50° overnight. 9-AMA then remains immobilized within in the TTAB phase of the mesoporous network. There was observed to be negligible leaching of 9-AMA from the particles, rapid response times and very good proton transport properties. For a full characterization of this material, see reference [20].

### Fluorescence Lifetime Measurements

In obtaining the FL of the immobilized acridine, fluorescence excitation was stimulated using sub-nanosecond pulses from a PicoQuant PLS light emitting diode with emission centered at 380 nm. The resulting fluorescence was collected using a PicoQuant PMA 175 photomultiplier, fitted with a 45 nm spectral filter centered at 452 nm. The photomultiplier response was digitized using a TimeHarp 260 Nano. The fluorescence lifetime values reported were determined by fitting exponential curves to the resulting fluorescence decay data using the FluoFit Pro software package.

### Fluorescence Emission Spectra

The emission spectra were obtained by illuminating samples using a Thorlabs LED with emission centered at 370 nm. The resulting fluorescence was collected and analyzed using a USB 2000 Ocean optics spectrometer, fitted with a 395 nm long pass filter.

## Results and Discussion

### 9-AMA

**Fig. 3:**
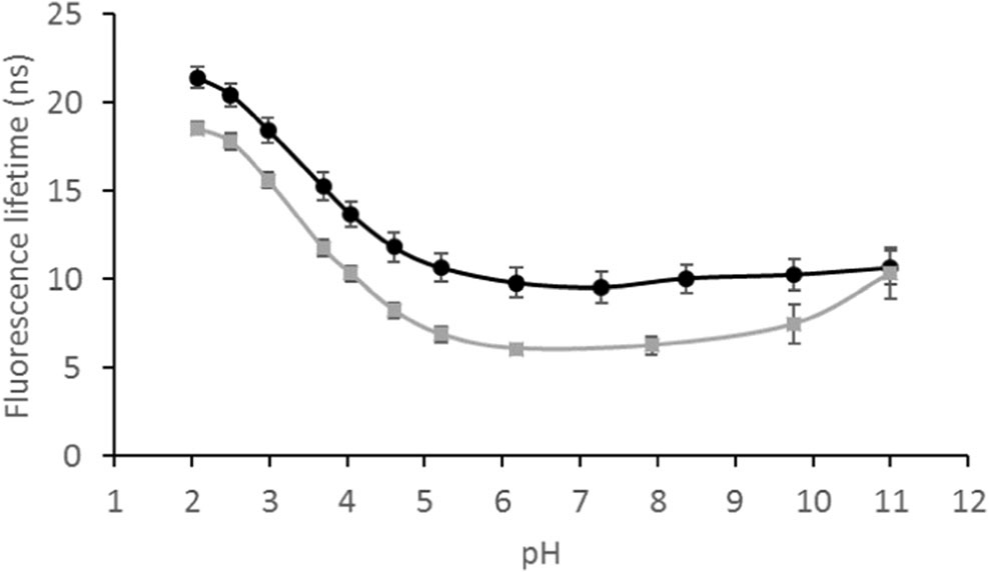
The fluorescence lifetime of 9-AMA versus pH dissolved in 100 mM buffer solutions () and immobilized in a surfactant-filled mesoporous silica (●)

Figure 3 shows FL versus pH for 9-AMA, both dissolved in 100 mM phosphate buffer and incorporated into the palisade region of a surfactant-filled mesoporous silica. Immobilization in the surfactant template of mesoporous silica has been shown to provide a stable chemical environment for FL fluorophores, negligible leaching, good proton transport properties, and a high loading capacity for the hydrophobic fluorophore [20]. Similar materials have successfully been used to host chemo-sensing molecules in general [26, 27].

The pK_a_ of 9-AMA is predicted at 7.8 (ACD/Labs Software). This is an expected value for the primary amine group of the molecule. However, the tertiary amine of 9-AMA, which is an integral part of the aromatic rings, can be expected to have a lower pK_a_. Thus, the 11 ns shift in FL between pH 2–5 is likely related to the tertiary amine. In comparison, the tertiary amine of the parent molecule of 9-AMA and 9-ACA, acridine, has a ground-state pK_a_ of 5.45 in aqueous solution, and the protonated and neutral form of acridine has a FL of 31.1 and 6.6 ns, respectively [18]. The increase in FL shows that the fraction of protonated 9-AMA increases sharply with a decrease in pH from 5 to 2, indicating a pK_a_ of around 3.5. Since the lone pair electrons of the tertiary amine occupy an sp^2^-orbital, conjugation with the π-electron system of the aromatic ring system is not possible; hence, they are readily accessible for protonation.

It is not surprising that protonation/deprotonation of the tertiary amine has a significant impact on the FL of 9-AMA, given the corresponding effect observed on the FL of acridine [18, 20]. The primary amine on the other hand, is located two bonds apart from the aromatic rings, and may as such not have a similar impact on the FL. At pH values above the expected pK_a_ of the primary amine (7.8), an increase in FL is observed, which is most pronounced above pH 9.8 where a 2.9 ns increase in FL occurs between pH 9.8–11. However, when the 9-AMA was incorporated into the surfactant phase of the mesoporous silica, the FL is nearly unchanged at pH values above 6. This is apparently an effect of the immobilization, and can be due to the orientation of 9-AMA in the surfactant palisade layer. Should the primary amine for example be located deeper into the surfactant layer, it will be less accessible to protonation.

**Fig. 4:**
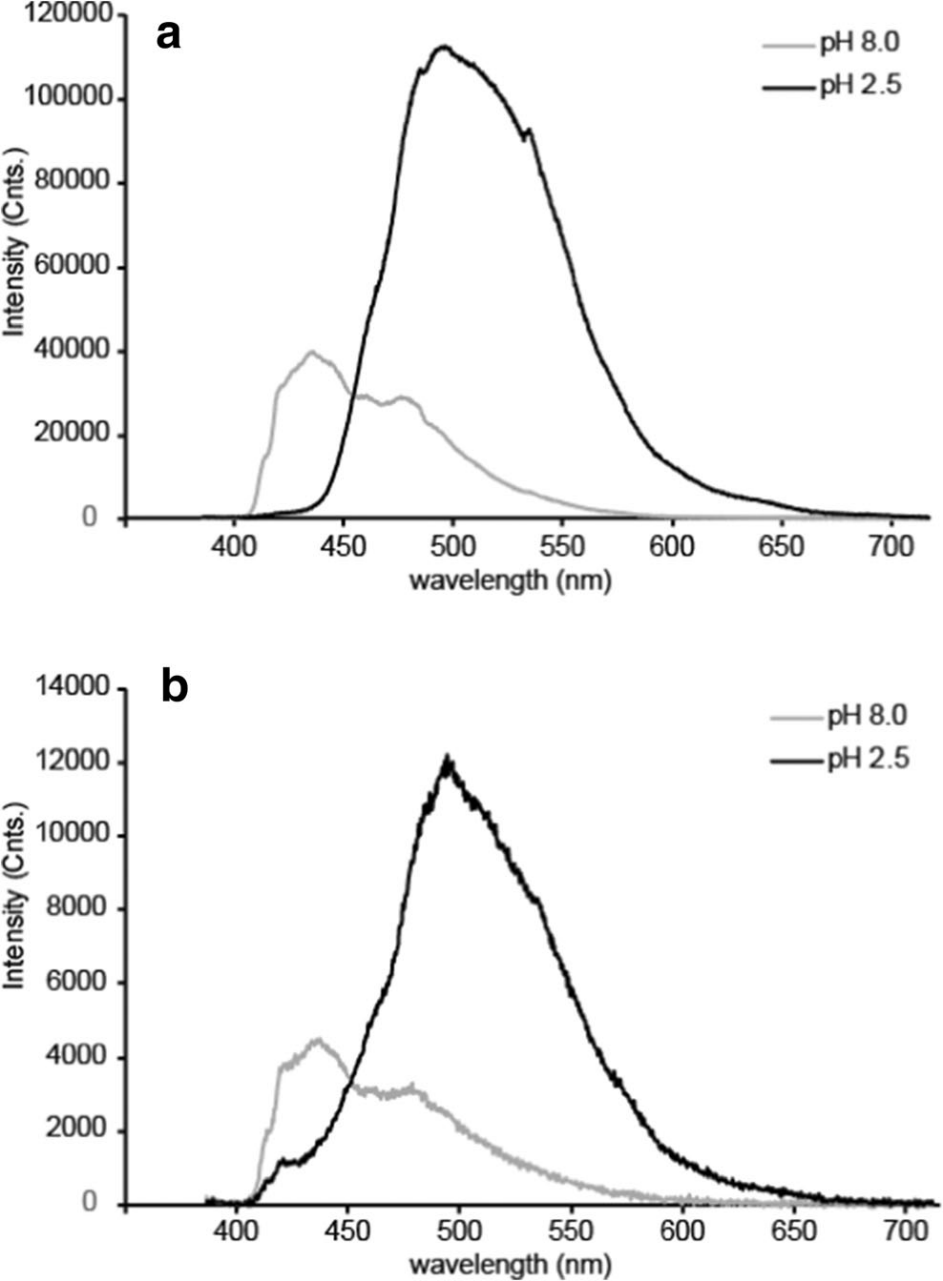
Emission spectra of 9-AMA dissolved in buffer solutions (**a**) and immobilized in mesoporous silica (**b**), at pH 8 and 2.5

Figure 4 shows the recorded spectra of 9-AMA in solution (A) and immobilized (B), at both high and low pH. In each case there is both a shift in emission wavelength and a significant reduction in intensity on going from low to high pH. At 500 nm emission, there is an 82% reduction in intensity from pH 2.5 to 8.0. In the same pH interval, there is a shift in the peak emission wavelength of about 75 nm. The red-shifted spectrum at low pH indicates presence of more resonance structures to stabilize the protonated molecule. Since the lone electron pair of the neutral tertiary amine belongs to an sp^2^ hybrid orbital, these do not contribute to the cyclic π-system and do not participate in resonance. This is likely the reason for this observation. In the neutral molecule, the C=N bond functions as an effective electron sink, causing various resonance structures to occur due to the partial positive charges created about the carbons in the ring. When protonated, an additional resonance structure occurs, which shifts the spectrum to longer wavelengths.

The figure shows that at 450 nm the intensity contributions for both the long and short lifetime species are approximately equal, justifying the choice of optical filters used when acquiring the FL data. The relatively modest change in the FL and fluorescence response properties of 9-AMA following immobilization is most likely due to the electrostatic nature of the indicator-immobilizer interaction, leaving the indicator’s internal electron transport properties relatively unchanged.

### 9-ACA

**Fig. 5:**
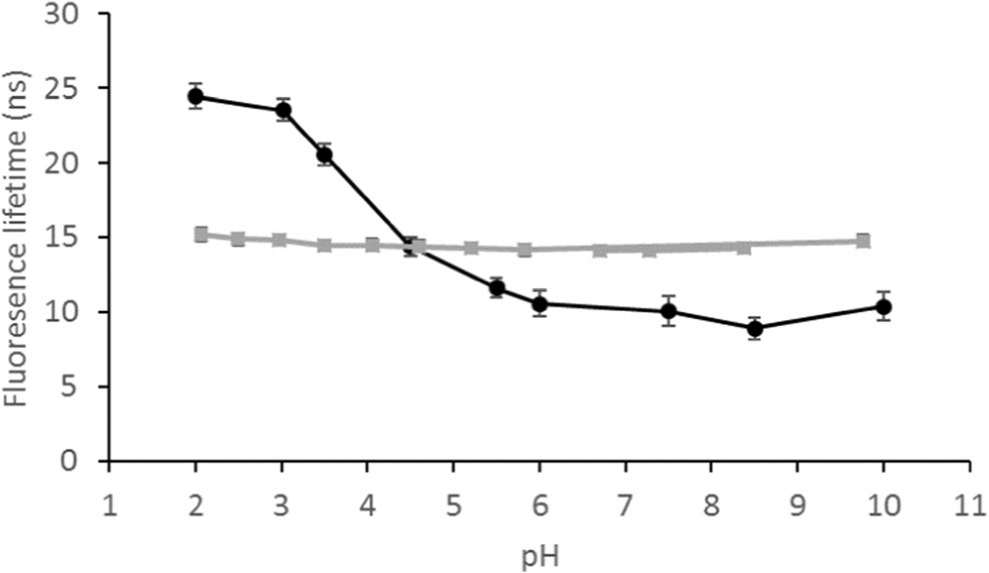
The fluorescence lifetime of 9-ACA versus pH dissolved in 100 mM buffer solutions () and covalently bound to amine-modified silica (●)

Figure 5 shows that pH has a negligible effect on the FL of 9-ACA. This can be due to the π-electrons of the C=O double bond interfering with those of the aromatic rings. However, when bound to the amine modified silica, the pH response characteristics changed dramatically, with the material exhibiting a FL decrease of 15 ns with increasing pH through the range pH 3–6.

**Fig. 6:**
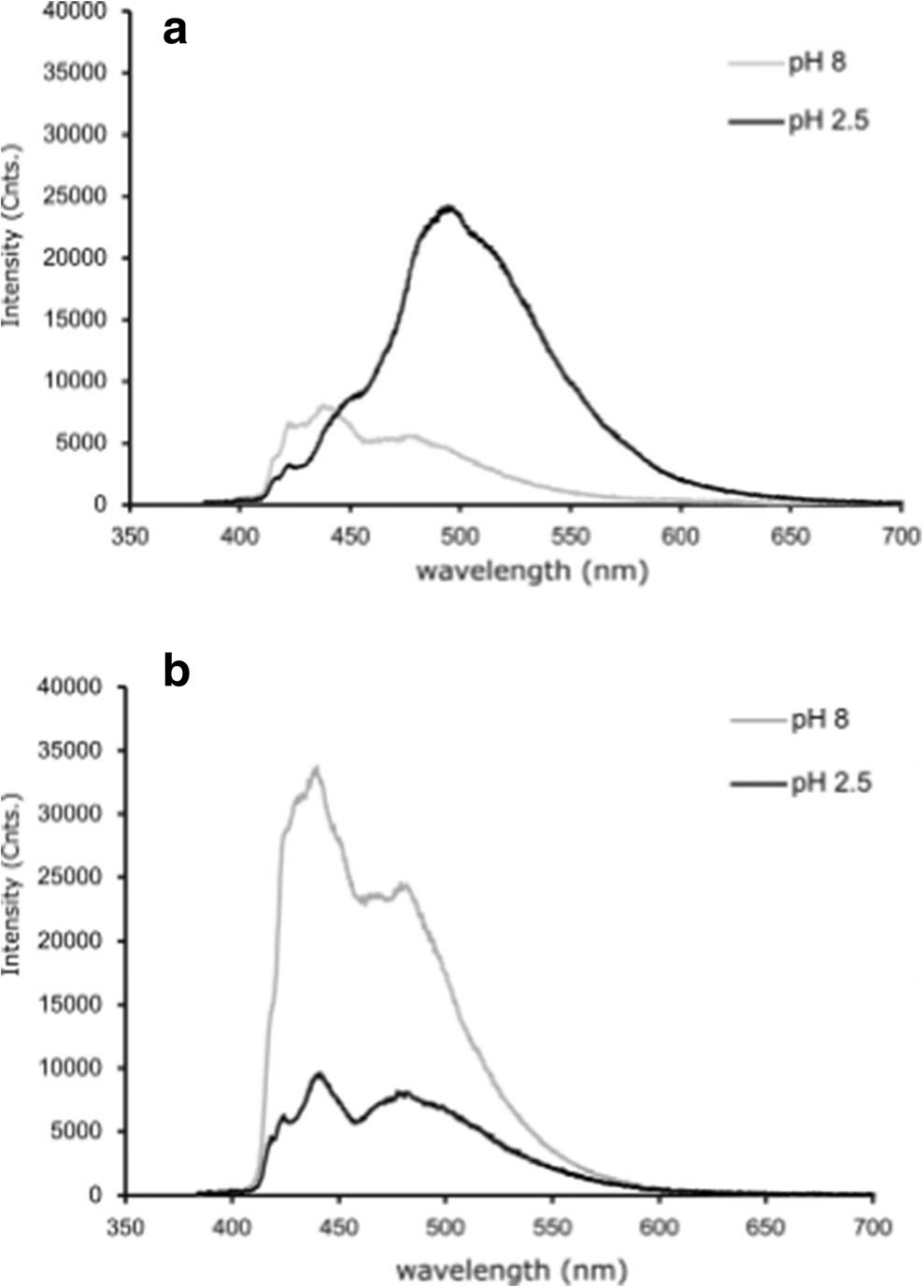
Emission spectra of 9-ACA covalently bound to silica (**a**) and dissolved in buffer solutions (**b**) and, at pH 8 and 2.5

Hence, in contrast to 9-AMA, the fluorescence spectrum of 9-ACA differs significantly for the buffer solubilized and immobilized cases (see Fig. 6). Immobilized 9-ACA exhibits a spectral shift to longer wavelengths with decreasing pH, similar to 9-AMA, whereas the solubilized 9-ACA shows no spectral shift. This indicates that the resonance structure attributed to the red-shifted spectrum at low pH does not occur in the buffer stabilized molecule, and it seems this resonance form is crucial for obtaining the good FL pH sensitivity. The dramatic change in the pH response behavior of both the fluorescence intensity spectrum and the FL for 9-ACA following immobilization is attributed to the effects of the 9-ACA – silica covalent bonds (see Fig. 2). During the process of surface bonding, the carbonyl (C=O) group adjacent to the aromatic rings is substituted with the less polar imine (C=N) group (Fig. 2). Such an alteration in polarity could explain the observed difference between the fluorescence properties of the surface bonded and buffer solubilized species. Moreover, imines are themselves mildly basic and will protonate at low pH. Thus, it is possible that the imine protonation contributes to the pH sensitivity of immobilized fluorophore fluorescence lifetime.

An advantage with this material is that the indicator is covalently bonded to the immobilizing matrix. This is convenient when considering long-term stability in a compact optical sensor, due to absence of e.g. the indicator leaching from or migrating within the immobilizing material.

Several fluorescence-based pH sensing principles have been proposed, where most rely on a change in intensity. This is caused by protonation/deprotonation of functional groups in close vicinity to the aromatic rings, which leads to partial quenching of the fluorescence. As mentioned, the motivation for using FL rather than intensity to monitor pH is that FL is an intrinsic property of an indicator, which is not affected by e.g. leaching, light scattering effects, excitation source intensity variations or photobleaching. Unfortunately, although many fluorophores have variations in fluorescence intensity with changes in pH, very few of these show significant FL pH sensitivity.

Acridine is amongst the indicators with highest fluorescence lifetime responses to pH [18–20], in the range of ~pH 5.5–8. Acridine immobilized in Nafion has previously been shown to be sensitive in the pH range between about 8.5–10.5 [18]. Thus, a higher pH FL indicator candidate to those presented here is available, although the important sensing range for oceanographic applications, between pH 8–8.5, remains a blind spot in terms of sensitive FL indicators. A previous publication indicated that diethylaminomethyl pyrene has potential as a sensitive fluorescence lifetime pH indicator in this pH range [28]. However, a major drawback with pyrene derived FL indicators is their high sensitivity to oxygen [29].

We recently showed that the FL pH sensing range of acridine can be extended by placing the fluorophore in a pH sensitive chemical environment, in this case an amine modified porous silica [12]. However, the total change in FL remains similar, and in order to achieve the highest possible sensitivity, it is an advantage to apply several sensitive indicators with different sensing ranges.

## Conclusions

9-ACA and 9-AMA complement acridine in widening the pH range where sensitive fluorescence lifetime indicators are available. These three indicators have overlapping sensing ranges from pH 2–8, which covers a broad range of applications. The pK_a_ of 9-AMA and immobilized 9-ACA lie at approximately 3.5, which to the best of our knowledge is lower than all other FL pH sensing fluorophores that have been demonstrated previously. With respect to the applicability of 9-ACA and 9-AMA as potential indicators in compact optical sensors, the long lifetime of these fluorophores makes it easier to delineate the signal from background fluorescence, which can be a considerable limitation for the application of fluorescence-based pH sensing [24]. Furthermore, we have demonstrated that both 9-ACA and 9-AMA can be successfully immobilized in robust substrates. Finally, the large FL shifts observed with pH, enables relatively simple optoelectronics to be used for their determination.
